# Implementing cancer prevention in occupational healthcare: initial insights from occupational healthcare staff in Central and Southern Europe – findings from the CPW project

**DOI:** 10.1186/s12885-026-15607-0

**Published:** 2026-01-22

**Authors:** Catharina Roth, Magdalena Kostrzewa, Giulia Collatuzzo, Valentina Biagioli, Monireh Sadat Seyyedsalehi, Stefano Giordani, Alessandro Godono, Marina Ruxandra Otelea, Dana Mates, Florina Georgeta Popescu, Adonina Tardon, Guillermo Fernandez-Tardon, Marta Maria Rodriguez-Suarez, Eleonóra Fabiánová, Jana Bérešová, Zuzana Klöslová, Miriam Kočtúchová Blažinová, Viktória Ďurajová, Iulia Crull, Anna Schneider-Kamp, Paolo Boffetta, Michel Wensing

**Affiliations:** 1 Department of General Practice and Health Services Research, Heidelberg University Hospital, Heidelberg, Germany; 2 Department of Medical and Surgical Sciences, University of Bologna, via Massarenti 9, Bologna, 40138 Italy; 3 Department of Biomedical and Clinical Sciences (DIBIC), Università degli Studi di Milano, Milano, 20122 Italy; 4 Onconauti Association, Metropolitan Oncology Network, via Paolo Nanni Costa 12/4A, Bologna, Italy; 5 Department of Public Health and Pediatrics, University of Torino, Piazza Polonia 94, Torino, 10126 Italy; 6 Colentina Clinical Hospital, Soseaua Stefan Cel Mare 19-21, Bucharest, 02012 Romania; 7 National Institute of Public Health, Dr. Leonte Anastasievici Street 1-3, District 5, Bucharest, 050463 Romania; 8 Clinical Department 5, University of Medicine and Pharmacy Carol Davila, Bucharest. 37, Dionisie Lupu St, Sector 2, Bucharest, 020021 Romania; 9 Health Research Institute of Principality of Asturias (ISPA), Avenida del Hospital, 33013, Oviedo and Faculty of Medicine, Universidad Nebrija, Campus Berzosa, Madrid, 28248 Spain; 10 Health Research Institute of Principality of Asturias (ISPA), and HUCA-SESPA, Oviedo, Asturias Spain; 11 Department of Occupational Health, Regional Authority of Public Health, Banská Bystrica, Slovakia; 12 Occupational Health Services, Železiarne Podbrezová a.s, Podbrezová, SK Slovakia; 13 F.D. Roosevelt Teaching Hospital with Policlinic Banska, Bystrica, Slovakia; 14 Romanian Society of Occupational Medicine, Bucharest, Romania; 15 Department of Business and Management, University of Southern Denmark, Campusvej 55, Odense M, 5230 Denmark; 16 Stony Brook Cancer Center, Stony Brook University, Stony Brook, NY USA; 17 Department of Family, Population and Preventive Medicine, Renaissance School of Medicine, Stony Brook University, Stony Brook, NY USA; 18 Heidelberg University, Medical Faculty, Im Neuenheimer Feld 130.3, Heidelberg, 69120 Germany

**Keywords:** Primary cancer prevention, Screening, Occupational health surveillance, Human papillomavirus, HPV, Hepatitis C virus, HCV, Helicobacter pylori, Hp, Workplace health program

## Abstract

**Background:**

Effective primary cancer prevention in occupational health care settings requires strategies tailored to workforce needs and individual risk profiles. Cultural, perceptual, and behavioural factors influence implementation success. Occupational healthcare professionals (OHCPs), with their expertise and regulatory responsibilities, are critical for advancing workplace cancer prevention. This study evaluates the feasibility of primary cancer prevention programs within the Cancer Prevention at Work (CPW) project across Europe from OHCP perspective.

**Methods:**

CPW is a Horizon Europe funded cross-sectional pilot study (2023–2026) conducted in Italy, Romania, Slovakia, and Spain. It focuses on HCV (Hepatitis C Virus) and Hp (Helicobacter pylori) screening and HPV (Human Papillomavirus) counselling among workers. OHCPs involved in program implementation completed a survey assessing their perceptions of the programs, contextual factors, and their professional role in delivery. Responses were recorded on a 5-point Likert scale (1 = strongly disagree, 5 = strongly agree), with higher scores indicating more positive assessments, supportive contexts, and greater OHCP engagement. Data were analysed using descriptive statistics and exploratory analyses.

**Results:**

Fifty-five OHCPs completed the survey. Findings suggest that integrating primary cancer prevention into occupational health is feasible. All three programs received positive evaluations (Mean Scores: 4.18–4.23; SD: 0.35–0.38), and organizational conditions, such as resources and leadership support, were rated favorably (Mean Scores: 4.24–4.73; SD: 0.42–0.61). OHCP involvement was moderately high (Mean Scores: 3.69–3.79; SD: 0.34–0.41), indicating meaningful engagement while highlighting opportunities for improvement. Assessments varied by setting and worker group, with more positive evaluations in hospitals and among healthcare or financial workers compared to metal workers. Contextual factors differed across groups, reflecting variability in perceived feasibility and ease of implementation.

**Conclusion:**

Conditions for the successful implementation of primary prevention programs targeting HCV, Hp, and HPV related cancers in occupational health services seem present. Particularly, if supported by favourable contextual factors and facilitated by employee participation. These findings offer preliminary evidence for the scalability of workplace-based cancer prevention strategies across diverse European health systems.

**Supplementary Information:**

The online version contains supplementary material available at 10.1186/s12885-026-15607-0.

## Background

Cancer prevention programs are crucial to reduce the global burden of cancer-related deaths [1]. These programs target the general population or specific subpopulations, supported by scientific research and local epidemiological evidence [1]. However, participation in many health promotion and disease prevention initiatives remains suboptimal, indicating that these efforts are not achieving their full potential [1]. A range of social, economic, and cultural factors pose barriers to successful implementation [2]. To address these issues, new avenues of cancer prevention should be introduced and promoted, particularly by identifying and targeting individuals and communities who are otherwise not reached. Since much of the adult population is employed, occupational healthcare services provide a promising opportunity to enact preventive strategies for cancer control. In this context, occupational medicine already addresses the risks associated with exposure to a variety of carcinogens, which might be present in the workplace [3–5]. The concept of integrating cancer prevention strategies into occupational medicine expands the focus beyond such work-related risks. Screening and vaccination for oncogenic infections are examples of such strategies and might have a profound impact, given that infection-related cancer amounts to 13% of the total world-wide cancer burden [6]. The benefits and costs of workplace-based cancer prevention programs need to be carefully verified before recommending widespread adoption. It has often been observed that the uptake of recommended practices is slow and incomplete, as widely documented in various contexts [7, 8]. Therefore, it is essential to consider the extent to which planned prevention interventions or prevention programs are adopted in practice, and how this can be enhanced.

The Cancer Prevention at Work (CPW) Project is an Europe-funded cancer prevention [9, 10]. The project focuses on three key prevention programs aimed at preventing cancers through screening for prevalent chronic infections and vaccinating against oncogenic infections: (i) screening for Helicobacter pylori (Hp) infection to prevent gastric cancer; (ii) screening for Hepatitis C Virus (HCV) to prevent liver cancer and non-Hodgkin lymphoma and other neoplasms; and (iii) counselling and vaccination against Human Papillomavirus (HPV) to prevent cervical, anogenital, and oropharyngeal cancers [8]. Human papillomavirus (HPV) and Hepatitis C Virus (HCV) infections are prioritized in cancer prevention programs due to their significant contribution to the European cancer burden and the effectiveness of vaccination and screening efforts [8]. Despite progress in cancer research, regional disparities persist, particularly in Central and Eastern Europe, where low vaccination uptake and limited access to screening underscore the critical need for strengthening these prevention strategies to reduce mortality and improve early detection across the continent [8]. Thus, these prevention programs are carried out in 8 implementation sites across four European countries: Italy, Spain, Romania, and Slovakia, targeting various types of workers.

From an implementation science perspective, the protocols for preventive interventions or prevention programs describe specific actions that need to be put into practice and scaled-up [11, 12]. Specific characteristics, such as the complexity of procedures, the visibility of the benefits, and the strength of the supporting evidence, can significantly influence the likelihood of these interventions being adopted by occupational healthcare providers [13, 14]. The cancer prevention strategies proposed within CPW are based on solid evidence, particularly concerning the risks associated with infectious agents such as Hp, HCV, and HPV [5, 15, 16]. Nevertheless, the efficiency of these primary prevention programs is influenced by the prevalence of the targeted infections within a specific population: the higher the prevalence of exposure to a particular risk factor, the more people will benefit and the more efficient the control of that risk factor will be [17]. This highlights the importance of tailoring strategies to match the burden of disease in different populations, ensuring that interventions are more impactful where the risk is more prevalent. Additionally, specific health-related perceptions, cultural beliefs, and behaviours of the targeted populations, i.e., workers in various organisations, play a significant role in realizing the health outcomes of the primary prevention programs [6, 17].

For the effectiveness of an occupational-based cancer prevention program, informed employees who are educated and proactive are crucial, along with the resources they need to pursue these goals [14]. Furthermore, the evaluation approaches employed by OHCPs, the contextual factors surrounding program implementation, and the extent of their involvement play a critical role in determining implementation outcomes. Additionally, behavioural and cultural factors influencing participation, such as willingness to engage in preventive measures, can also affect how OHCPs implement these interventions. For example, low worker participation may diminish OHCPs’ and employers’ motivation to offer preventive programs [18]. Therefore, this study aimed to (I) assess the feasibility of three primary prevention programs by OHCPs involved in the implementation and delivery of the prevention programs in Italy, Romania, Slovakia, and Spain and to (II) explore contextual factors affecting implementation and to (III) examine OHCPs’ views on their roles in the process.

## Methods

### Study design

#### Main study

The primary prevention program(s) were implemented as a prospective pilot study in various occupational health surveillance (OHS) and workplace settings in 8 implementation centres in Italy, Romania, Slovakia, and Spain, and targeted a minimum of 1000 workers in each participating country and implementation centre (Table 1). Participant enrollment began between May 2024 and January 2025 and is projected to continue for up to 24 months, likely concluding by April 2026 or once recruitment goals are met. Details on the CPW protocol have been previously published [10].

**Table 1:** Overview of the implementation centre

Centre	Country	Intervention	Start of the pilot Study	Organisation Type	Targeted Workers^**^
1	Spain	Hp Screening	June 2024	Non-profit organisation	Service and Manufacturing Workers
2	Romania	Hp Screening	June 2024	Hospital	Healthcare Workers
3	Romania	HCV Screening	June 2024	Hospital	Healthcare Workers
4	Slovakia^*^	HCV Screening	October 2024	Hospital	Healthcare Workers
5	Slovakia^*^	HPV Counselling	May 2024	Public Health Institute	Healthcare Workers
6	Slovakia^*^	Hp Screening	September 2024	Small and medium-sized enterprises	Metal Workers
7	Italy	Hp Screening	January 2025	Non-profit organisation	Service and Service and Manufacturing Workers
		HCV Screening			
8	Italy	HCVScreening	October 2024	Large Company	Financial Workers
		HPV Counselling			

^*^Slovak centers only conduct serological testing of Hp antibodies (IgA, IgM, IgG) to assess prevalence, provide results to respondents’ General Practitioners (GPs), and leave further diagnostics to GPs according to Ministry of Health guidelines

^**^Across all implementation sites, the total number of workers included per intervention at the time of data collection follows: 3,000 for Hp screening, 5,000 for HCV screening, and 3,000 for HPV counselling

#### Process evaluation

To evaluate the implementation of the primary prevention program(s), a process evaluation was conducted alongside the main study [10] to examine the (I) feasibility of the prevention programs by OHCPs involved in the implementation process and the delivery of the prevention program(s), (II) to explore contextual factors, and to (III) examine OHCP’s roles in the process. A questionnaire based on the Consolidated Framework for Implementation Research (CFIR) by Damschroder [19] was developed and translated into each language (Italian, Spanish, Romanian, and Slovakian). The questionnaire was designed to be repeated two times: (i) at the very start of the implementation period (baseline questionnaire – T0) and (ii) at the end of the implementation period (follow-up questionnaire – T1). The distribution of the survey was managed by the local research teams. Due to the rotation of healthcare personnel across different centers, a comparative analysis linking T0 and T1 will be feasible for a subgroup of healthcare providers participating to both surveys. This would enable analyses of potential changes in responses over time. This manuscript focuses on the baseline questionnaire (T0).

#### Description of the primary prevention program(s)

**Fig. 1:**
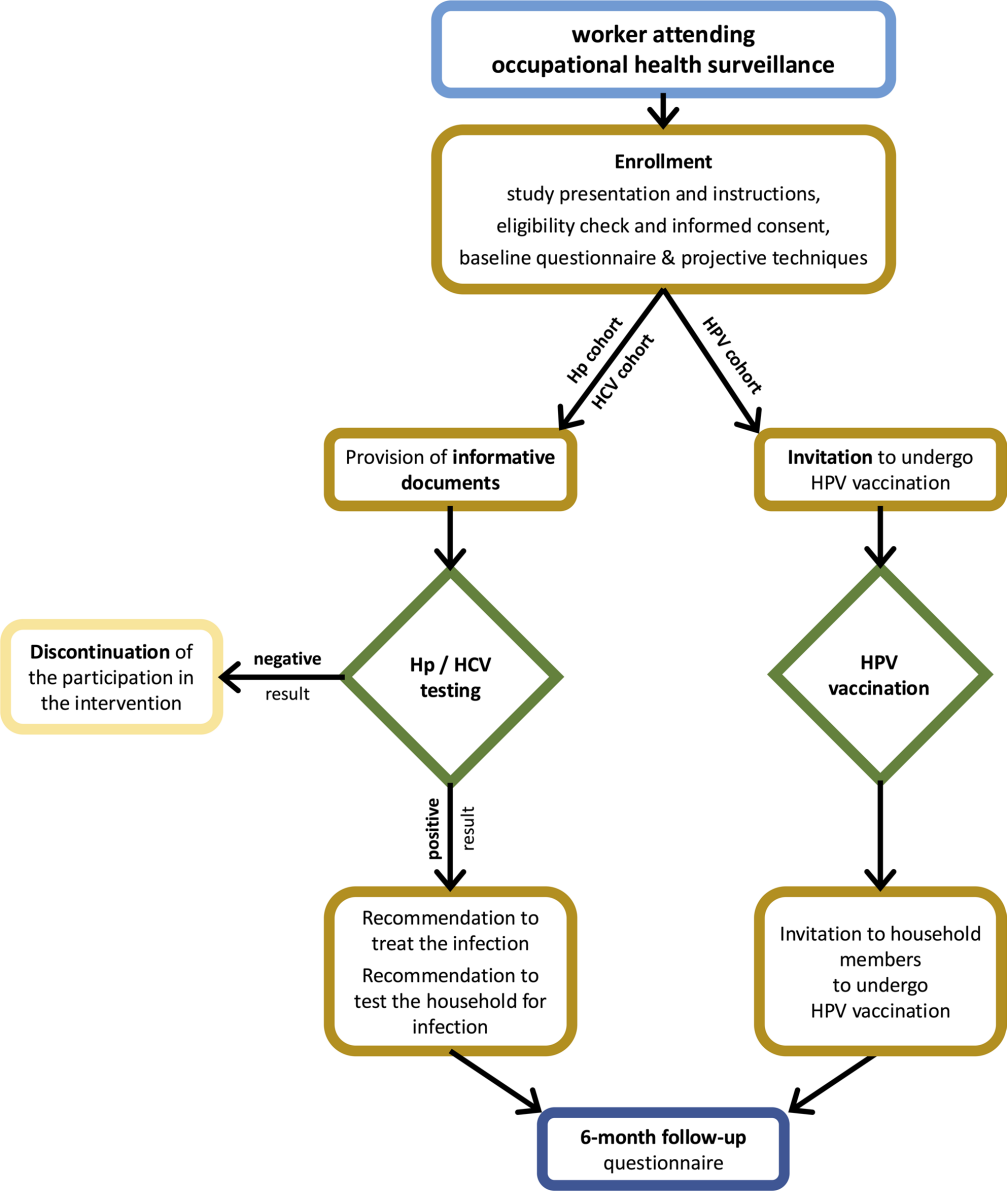
Schematic overview of the CPW preventive programs. (adapted from Collatuzzo et al. [20])

Each program involved testing for Hp, and/or HCV, and/or counselling on HPV vaccination. Workers who tested positive confirmed for Hp or HCV infections are counselled about their condition with treatment options and referred to appropriate healthcare services. Workers who participated in the counselling on HPV vaccination received informative material and were offered to be vaccinated after providing consent according to standard protocols based on the intention to treat. They were also followed up at six months through another questionnaire. The workers who participated in each prevention program received informative material about the available strategies to prevent cancer related to these infections. In addition, workers who tested positive for Hp or HCV were advised on testing and treatment options for their adult first-degree relatives and household members. Similarly, workers involved in the HPV vaccination intervention were offered the opportunity to have their eligible family members vaccinated as well. Figure 1 summarises the established protocol for the preventive programs in the CPW project.

#### Implementation strategies

Several implementation strategies were applied to promote the successful implementation of the three prevention programs. Implementation strategies were mapped to the Expert Recommendation for Implementing Change (ERIC) Taxonomy [21] and adapted to the CPW project. The ERIC Taxonomy provides a comprehensive compilation of implementation strategies designed to support the systematic implementation of evidence-based interventions. Some strategies aim to directly change healthcare practice (e.g., training of occupational healthcare professionals), while others support these changes, yet do not induce change by themselves [21]. Additionally, plans were developed for large-scale implementation within OHS and broader public health networks, aiming to extend the reach of the prevention programs beyond the CPW project (Table 2).

**Table 2:** Overview of implementation strategies in the CPW project

Implementation Strategy	ERIC Category [21]	Timing in relation to prevention intervention in CPW project
Identify and build on existing high-quality working relationships and network within and outside the organization	Promote network weaving	Before & During
Hold meetings targeted toward different stakeholder groups to inform about the prevetion programs	Conduct educational meetings	Before & During
Identify or start a separate organization that is responsible for disseminating the prevention programs.	Start a dissemination organization	Before
Distribute educational material including guidelines, manuals, brochures, posters, and toolkits in person, by mail, and/or electronically	Distribute educational material	Before
Use a survey to assess barriers for implementation from the perspective of occupational healthcare professionals	Assess for readiness and identify barriers and facilitators	During: early and late in the implementation phase
Develop plans for large-scale implementation within OH surveillance and clinical and public health system.	Develop a formal implementation blueprint	After
Identify and engage potential stakeholders and network in support of occupational-based cancer prevention programs	Promote network weaving	Before, During, After

### Participants

All OHCPs (older than 18 years) at each implementation center across the participating countries who were involved in the implementation and delivery of the prevention programs(s) within the CPW project were invited to participate in the survey. Participation was voluntary. Informed consent was implied through the completion of the questionnaire.

### Data collection

Data was collected through a paper-based survey administered to OHCPs. The survey was conducted by the local research teams four to eight weeks after the local start of the pilot study at each implementation center. All occupational healthcare providers involved in the delivery of the intervention were invited to participate in the survey. The local start of the pilot study varied slightly by implementation site, based on the beginning of interventions implementation. Prior to the local start of the study, the questionnaire items were translated into the local language by members of the local research teams who were fluent in English and the local language, and then back-translated to ensure accuracy.

### Measurements

The first section of the questionnaire included sociodemographic questions (11 items). Possible answer options were provided. The second section of the questionnaire was divided into three different domains (Supplementary Material 1): Assessment of the Prevention programs (I), Contextual Factors Influencing the Implementation of the Prevention Programs (II), and Role of OHCP during the Implementation of the Prevention Programs (III) (Table 3). Response options included a 5-point Likert Scale from 1 (“strongly disagree”) to 5 (“strongly agree”) and two additional answering options (Don’t Know and Not Applicable) for each part. A detailed description of the questionnaire can be found in Supplementary Material 1.

**Table 3:** Summary of occupational healthcare provider questionnaire used in the CPW project

Domain		Assessment Method
First Section		
I	Characteristics of participating OHCP (11 items)	Newly developed questions developed for the specific prevention program(s)
Second Section		
I	Assessment of the Prevention Program (6 items)	Newly developed questions developed for the prevention program(s) based on the CFIR questionnaire (19)
II	Contextual Factors influencing the Implementation of the Prevention Program (10 items)	Newly developed questions developed for the prevention program(s) based on the CFIR questionnaire (19)
III	Role of OHCP during the Implementation of the Preventon Program (e.g.; nurses or physicians depending on the center) (9 items)	Newly developed questions developed for the prevention program(s) based on the CFIR questionnaire (19)

#### Domain I: assessment of the prevention program

This part of the questionnaire was used to evaluate how OHCPs assessed the three prevention programs. Including if the prevention programs came from a reliable source (Item 1), if implementation of the prevention programs outweighs the disadvantages regarding health improvements (Item 2), and if the research evidence for the new prevention programs is sufficiently strong (Item 3). In addition, if OCPHs saw potential harm in the implementation of the three prevention programs (Item 4), if the provision of the programs is complex (Item 5), and if the provision is sufficiently flexible in practice (Item 6). Higher scores indicate a more positive assessment, while lower scores suggest a more negative assessment of the three prevention programs.

#### Domain II: contextual factors influencing the implementation of the prevention program

This part was used to evaluate barriers and facilitators that may influence the implementation of the prevention program(s). It included items assessing whether sufficient time was available to implement the program(s) (Item 1) and whether the workplace was prepared (Item 2). Furthermore, it examined if information for workers was well-organized (Item 3), and whether both the team leader (Item 4) and the employer(s) (Item 5) supported the new prevention programs. Additional items assessed the organization of follow-up procedures (Item 6), the prioritization of the prevention program(s) (Item 7), the general speed of implementing new programs (Item 8), the support from local opinion leaders (Item 9), and the availability of additional support (Item 10). Higher scores indicate more favourable organizational conditions for implementing the prevention program(s), while lower scores reflect less favourable conditions.

#### Domain III: OHCP role during the implementation of the prevention programs

This part assessed various professional attitudes and perceptions regarding the three prevention programs. Participants reported on the alignment of their professional attitudes with the programs’ basic principles (Item 1) and their involvement in the implementation process from the project’s outset (Item 2). They also evaluated their perceived competence to implement the programs (Item 3) and their ability to exclude individual workers from participation for medical reasons (Item 4). The assessment included perceptions of resistance from workers (Item 5) and whether colleagues influenced their decision to implement the programs (Item 6). Additionally, participants indicated their capability to adapt daily operational procedures to accommodate the new programs (Item 7), feelings of being overwhelmed by the changes (Item 8), and the extent to which the programs substantially altered their work tasks or responsibilities (Item 9). Higher scores reflect a higher level of OHCP engagement in the implementation of the three prevention programs, whereas lower scores may indicate limited or inconsistent involvement.

### Psychometric evaluation

The questionnaire was developed using the Consolidated Framework for Implementation Research (CFIR) as a guiding framework [19]. To assess its internal consistency, both Cronbach’s alpha and Omega coefficients were calculated. Furthermore, the questionnaire underwent multiple rounds of review and revisions among the developing research team, the coordinating team, and research team members at the implementation centers. This iterative process continued until a consensus version was achieved.

### Data analysis

All available datasets were included. Prior to analysis, variables were screened for data entry errors and missing values. The three domains (Table 2) were measured using 5-point Likert scales, with varying numbers of items per domain. Items belonging to the same domain were aggregated into Mean Scale Scores to create composite variables. This approach is widely applied in survey research to reduce measurement error and more reliably capture the underlying latent domains [22]. Response options “Do not know” and “Not applicable” were treated as user-defined missing values [23] and negatively worded items were reverse-coded in order to include them in the composite variables. Descriptive statistics (means and standard deviations for continuous variables; frequencies and percentages for categorical variables) were calculated. Cronbach’s alpha and Omega were calculated to assess the internal consistency for composite variables. Acceptable internal consistency was set at Cronbach’s alpha ≥ 0.80 and Omega ≥ 0.70 (Supplementary Material 2). Cronbach’s alpha indicates a lower bound of internal consistency under the assumption of tau-equivalence, meaning that all items within a domain contribute equally to the construct being measured. In contrast, Omega is a more general index of reliability, accounting for the hierarchical structure of the data and allowing for unequal contributions from each item within a domain [24, 25]. In addition to the composite variables, individual items were examined separately because some items were considered particularly clinically relevant. This allowed for a more detailed analysis of specific aspects of the domains.

To examine differences in the dependent variables: (I) Assessment of the Prevention Program, (II) Contextual Factors, and (III) Role of OHCP, a One-Way Analysis of Variance (ANOVA) was conducted across six sociodemographic variables (Gender, Age Group, Professional Background, Years in Practice, and Years in current Medical Team) and four variables characterising the implementation centres (Occupational Healthcare Setting, Workers Categories, and Workers Reached by Prevention Program). Due to limited sample sizes within countries, country-specific analyses were not conducted. Additionally, the results of the One-Way ANOVA should be interpreted with caution, as some groups included very small sample sizes. All analyses were performed using IBM SPSS Statistics, Version 25 [26]. Statistical significance was set at *p* < 0.05.

### Ethical considerations

The study has been approved by the local ethics committees at each implementation centre and the coordination centre. Participation in the paper-based survey was anonymous and voluntary. The completion of the questionnaire was considered consent to participate in the OHCP survey of the CPW project. Research in this study was conducted in accordance with the Declaration of Helsinki.

## Results

### Description of the study population

The first part of the questionnaire was used to describe the study population. In total 55 OHCPs across the four countries involved in the CPW project participated in the paper-based survey (Response Rate = 96.5%). The majority of the participating OHCPs identified as female (76.4%). Half of them were over 50 years old (50.9%). A majority held positions as physician (58.2%) and had over 20 years of professional experience (54.5%). Less than half had been part of the current medical team for more than five years (41.8%). A detailed description of the study population can be found in Table 4.

**Table 4:** Description of the main characteristics of the study population

Sociodemographic Variables	*N* = 55 (100%)
Occupational Healthcare Provider per Countries	
Italy	13 out of 13 (23.6)
Spain	10 out of 12 (18.2)
Romania	14 out of 14 (25.5)
Slovakia	18 out of 18 (32.7)
Gender	
Identify as Female	42 (76.4)
Identify as Male	13 (23.6)
Age Groups	
below 30 years	2 (3.6)
30–39 years	10 (18.2)
40–49 years	15 (27.3)
50 + years	28 (50.9)
Professional Background	
Physicians (including Resident Physicians)	35 (63.6)
Nurses	10 (18.2)
Other Health Specialists (e.g., occupational or public health specialsists)	10 (18.2)
Years in Practice	
Less than 10 years	19 (34.5)
10–19 years	6 (10.9)
20 + years	30 (54.5)
Years in Current Medical Team	
Less than 1 year	14 (25.5)
1–4 years	18 (32.7)
5 + years	23 (41.8)

The participating occupational healthcare providers were involved in the delivery of the prevention programs within various occupational health settings: hospitals, small and medium-sized enterprises, public health institutes, and large companies. Almost half of the targeted workers were healthcare workers (49.4%), with smaller groups from metal workers, manufacturing, financial sectors, and others. The prevention program(s) offered included either Hp screening, HCV screening, and/or HPV Counselling, with the most common combination being offered all three interventions (32.7%). Most of the participating OHCP had delivered the prevention program(s) to more than 50 workers on average at the time of the questionnaire (72.7%). A detailed description can be found in Table 5.

**Table 5:** Description of the context of the implementation centre

Characteristics of the Implementation Centres	*N* = 55 (100%)
Occupational Health Settings	
Hospitals	22 (40.0)
Small & Medium-sized Enterprises	8 (14.5)
Public Health Institutes	9 (16.4)
Large Companies	16 (29.1)
Targed Workers	
Healthcare Workers	27 (49.1)
Metal Workers	5 (9.1)
Manufacturing Workers	6 (10.9)
Financial Workers	5 (9.1)
Retail Workers	2 (3.6)
Others^*^	10 (18.2)
Prevention Program(s)	
Hp Screening	15 (27.3)
HCV Screening	9 (16.4)
Hp and HCV Screening	8 (14.5)
HCV Screening and HPV Counselling & Vaccination	5 (9.1)
Hp and HCV Screening and HPV Counselling & Vaccination	18 (32.7)
Workers reached by the Prevention Program	
0–9 workers	7 (12.7)
10–49 workers	8 (14.6)
More than 50 workers	40 (72.7)

^*^Other targeted workers can include administrative staff, mechanics, hospital bar employees, coffee pickers, vehicle maintenance personnel, and drivers

#### Domain I: assessment of the prevention programs

OHCPs assessed the three different prevention programs as highly positive (Mean Scale Scores 4.18 to 4.23), indicating that the implementation of these programs is considered sufficiently feasible in practice. OHCPs believe the prevention programs originate from a reliable source (Mean Scores 4.74 to 4.91) and regard the research evidence supporting them as strong (Mean Scores 4.50 to 4.67). Furthermore, participants perceived that the new prevention programs offer significant advantages in terms of health improvements (Mean Scores 4.69 to 4.85) (Table 6). Participating OHCPs did not perceive any harm associated with implementing the three new prevention programs within their occupational health services (Mean Scores 1.59 to 2.33). Additionally, the results suggest that the implementation of the primary prevention programs is flexible (Mean Scores 3.95 to 4.31) and can be easily integrated into the workflow of occupational health services (Mean Scores 2.68 to 3.17) (Table 6).

**Table 6:** Domain I: assessment of the prevention programs

	*N* = 55	Min	Max	Mean Score (SD)
Scale Score: HCV Screening^*^	40	3.0	5.0	4.20 (0.38)
Program comes from a reliable source^*^	40	3.0	5.0	4.77 (0.67)
Program has many advantages^*^	40	4.0	5.0	4.73 (0.45)
Program is based on strong evidence^*^	39	3.0	5.0	4.67 (057)
The program is harmful^*^	39	1.0	5.0	2.05 (0.92)
Provision of the program is flexible^*^	39	3.0	5.0	4.31 (0.65)
Provision of the program is complex^*^	39	1.0	5.0	2.77 (1.22)
Scale Score: Hp Screening^*^	40	3.2	4.8	4.18 (0.35)
Program comes from a reliable source^*^	39	3.0	5.0	4.74 (0.64)
Program has many advantages^*^	39	3.0	5.0	4.85 (0.43)
Program is based on strong evidence^*^	40	2.0	5.0	4.50 (0.75)
The program is harmful^*^	39	1.0	5.0	2.33 (1.34)
Provision of the program is flexible^*^	39	1.0	5.0	4.13 (0.83)
Provision of the program is complex^*^	40	1.0	5.0	3.17 (1.29)
Scale Score: HPV Counselling^*^	23	3.5	5.0	4.23 (0.35)
Program comes from a reliable source^*^	23	4.0	5.0	4.91 (0.28)
Program has many advantages^*^	23	4.0	5.0	4.69 (0.47)
Program is based on strong evidence^*^	23	3.0	5.0	4.61 (0.66)
The program is harmful^*^	22	1.0	4.0	1.59 (0.79)
Provision of the program is flexible^*^	23	2.0	5.0	3.95 (0.87)
Provision of the program is complex^*^	22	1.0	5.0	2.68 (1.08)

^*^Higher scores indicate that OHCP tended to agree with the statements, while lower scores indicate disagreement; Scale used: 1 (Strongly Disagree) − 5 (Strongly Agree); Number of Items included: 6

#### Domain II: contextual factors influencing the implementation prevention programs

Participating OHCP rated the contextual factors influencing the implementation of the three prevention programs positively, with Mean Scale Scores ranging from 4.24 to 4.37 (Table 6). These high ratings indicate that OHCP felt their workplaces were well-equipped to support the new initiatives (Mean Scores 4.32 to 4.61). Additionally, OHCP reported having sufficient time in their daily practice to implement the programs (Mean Scores 3.74 to 4.15). Participants also perceived that the programs received adequate priority within their workplaces (Mean Scores 4.20 to 4.41) (Table 7). Follow-up procedures (if needed) were viewed as well-structured (Mean Scores 4.31 to 4.33), and information regarding the new programs was generally well-organized (Mean Scores 4.05 to 4.35). Strong support was reported from both the heads of the occupational healthcare teams (Mean Scores 4.76 to 4.83) and the employers (Mean Scores 4.71 to 4.83). Furthermore, support from local opinion leaders such as occupational health physicians, safety and health advisors, coordinators of health surveillance programs, and other seior healthcare professionals (Mean Scores 3.76 to 4.09) and the organisation (e.g., the hospitals or the public health institutes) itself (Mean Scores 3.95 to 4.33) was positively perceived. OHCP also noted that their workplaces had a history of implementing new programs efficiently (Mean Scores 3.90 to 4.04) (Table 7).

**Table 7:** Domain II: contextual factors influencing the implementation of the prevention programs

	*N* = 55	Min	Max	Mean Score (SD)
Scale Score: HCV Screening^*^	40	2.7	5.0	4.24 (0.61)
Sufficient time to deliver the program^*^	38	2.0	5.0	3.84 (1.07)
Readiness to implement the program^*^	40	2.0	5.0	4.45 (0.71)
Information on the program is organised^*^	40	2.0	5.0	4.32 (0.76)
Teamleader supports the program^*^	40	4.0	5.0	4.80 (0.41)
Employer support the new program^*^	38	3.0	5.0	4.71 (0.46)
Follow up screening is well organised^*^	32	3.0	5.0	4.31 (0.69)
Delivery of the program has priority^*^	40	2.0	5.0	4.20 (0.82)
Implementation of the new program is fast^*^	30	2.0	5.0	3.90 (0.92)
Local opinion leader support the program^*^	35	2.0	5.0	3.76 (0.69)
Organisation provides additonal support^*^	38	2.0	5.0	3.95 (0.95)
Scale Score: Hp Screening^*^	41	3.6	5.0	4.37 (0.42)
Sufficient time to deliver the program^*^	41	3.0	5.0	4.15 (0.65)
Readiness to implement the program^*^	41	1.0	5.0	4.05 (0.86)
Information on the program is organised^*^	41	3.0	5.0	4.61 (0.54)
Teamleader supports the program^*^	41	3.0	5.0	4.76 (0.49)
Employer support the new program^*^	41	4.0	5.0	4.71 (0.46)
Follow up screening is well organised^*^	37	3.0	5.0	4.31 (0.69)
Delivery of the program has priority^*^	41	3.0	5.0	4.41 (0.67)
Implementation of the new program is fast^*^	24	2.0	5.0	4.04 (0.99)
Local opinion leader support the program^*^	33	3.0	5.0	4.09 (0.68)
Organisation provides additonal support^*^	36	3.0	5.0	4.33 (0.75)
Scale Score: HPV Counselling^*^	23	3.6	5.0	4.24 (0.47)
Sufficient time to deliver the program^*^	23	2.0	5.0	3.74 (0.81)
Readiness to implement the program^*^	23	3.0	5.0	4.35 (0.65)
Information on the program is organised^*^	23	3.0	5.0	4.35 (0.65)
Teamleader supports the program^*^	23	4.0	5.0	4.83 (0.39)
Employer support the new program^*^	23	4.0	5.0	4.83 (0.39)
Follow up screening is well organised^*^	15	3.0	5.0	4.33 (0.62)
Delivery of the program has priority^*^	23	3.0	5.0	4.22 (0.59)
Implementation of the new program is fast^*^	22	2.0	5.0	3.82 (1.05)
Local opinion leader support the program^*^	20	2.0	5.0	3.90 (0.78)
Organisation provides additonal support^*^	23	2.0	5.0	4.04 (1.06)

^*^Higher scores indicate that OHCP tended to agree with the statements, while lower scores indicate disagreement; Scale used: 1 (Strongly Disagree) − 5 (Strongly Agree); Number of Items included: 10

#### Domain III: OHCP role during implementation of the prevention programs

OHCP rated their engagement during the implementation of the prevention programs as neutral to moderate (Mean Scale Scores 3.69 to 3.79). This suggests that, while they recognised their involvement, they felt their engagement was neither particularly high nor low. Participating occupational healthcare provider indicated that their professional attitudes aligned with the basic principles of the new programs (Mean Scores 4.30 to 4.50), and they reported feeling involved from the beginning of the implementation process (Mean Scores 4.19 to 4.57). OHCP felt sufficiently competent to implement the new screening or counseling programs (Mean Scores 4.16 to 4.35). OHCP perceived little resistance from workers (Mean Scores 2.51 to 2.77), although there was considerable variability in responses (SD 0.88 to 1.11) (Table 8). OHCP were mostly neutral, with some variation in opinions about excluding workers for medical reasons (Mean Scores 2.94 to 4.00; SD 1.05 to 1.32). They generally agreed that colleagues convinced them to implement the new programs, although there was some variation in responses (Mean Scores 3.22 to 3.33; SD 1.10 to 1.13). Additionally, most OHCP felt capable of adapting their operational procedures to implement the programs (Mean Scores 3.85 to 4.07) (Table 8). Overall, OHCP seemed to feel capable and not overwhelmed by the changes related to the implementation of the new programs (Mean Scores 2.39 to 2.42). Furthermore, participants generally did not feel that the implementation of the new programs substantially changed their areas of work (Mean Scores 2.77 to 3.13) (Table 8).

**Table 8:** Domain III: OHCP role during implementation of the prevention programs

	*N* = 55	Min	Max	Mean Score (SD)
Scale Score: HCV Screening^*^	40	3.0	4.8	3.77 (0.41)
Professional attitude matches with program^*^	40	3.0	5.0	4.50 (0.65)
Involvement from the beginning^*^	40	3.0	5.0	4.57 (0.63)
Sufficiently competend to deliver program^*^	40	2.0	5.0	4.35 (0.83)
Exclusion due to medical reasons^*^	39	1.0	5.0	3.21 (1.32)
Perceived Resistance from workers^*^	39	1.0	4.0	2.51 (0.88)
Colleagues convinced me to implement the program^*^	27	1.0	5.0	3.33 (1.11)
Ability to change operational procedures^*^	39	2.0	5.0	3.85 (0.87)
Feeling overstrained by changes^*^	40	1.0	4.0	2.42 (0.71)
Changes of area of work due to the program^*^	40	1.0	5.0	2.87 (1.06)
Scale Score: Hp Screening^*^	41	3.1	4.3	3.79 (0.34)
Professional attitude matches with program^*^	41	3.0	5.0	4.48 (0.59)
Involvement from the beginning^*^	41	1.0	5.0	4.19 (1.21)
Sufficiently competend to deliver program^*^	41	1.0	5.0	4.36 (0.83)
Exclusion due to medical reasons^*^	39	1.0	5.0	4.00 (1.15)
Perceived Resistance from workers^*^	39	1.0	5.0	2.77 (1.11)
Colleagues convinced me to implement the program^*^	41	1.0	5.0	3.22 (1.13)
Ability to change operational procedures^*^	40	2.0	5.0	4.07 (0.82)
Feeling overstrained by changes^*^	41	1.0	4.0	2.36 (0.94)
Changes of area of work due to the program^*^	40	1.0	5.0	2.77 (1.18)
Scale Score: HPV Counselling^*^	23	2.8	4.6	3.69 (0.41)
Professional attitude matches with program^*^	23	3.0	5.0	4.30 (0.76)
Involvement from the beginning^*^	23	4.0	5.0	4.52 (0.51)
Sufficiently competend to deliver program^*^	23	3.0	5.0	4.16 (0.68)
Exclusion due to medical reasons^*^	18	1.0	5.0	2.94 (1.05)
Perceived Resistance from workers^*^	22	1.0	5.0	2.77 (1.11)
Colleagues convinced me to implement the program^*^	17	1.0	5.0	3.29 (1.10)
Ability to change operational procedures^*^	22	2.0	5.0	3.72 (0.82)
Feeling overstrained by changes^*^	23	1.0	4.0	2.39 (0.89)
Changes of area of work due to the program^*^	23	1.0	5.0	3.13 (1.09)

^*^Higher scores indicate that OHCP tended to agree with the statements, while lower scores indicate disagreement; Scale used: 1 (Strongly Disagree) − 5 (Strongly Agree); Number of Items included: 9

### One-way analysis of variance (ANOVA)

A one-way analysis of variance (ANOVA) was conducted to examine differences between sociodemographic variables, variables characterising the implementation centres, and the three outcome measures: I Assessment of the Prevention Program, II Contextual Factors Influencing the Implementation of the Prevention Programs, and III Role of OHCP during the Implementation of the Prevention Programs. Only significant differences between the outcome measures and these variables are presented below:

### Hp screening

#### Assessment of the Hp screening

The ANOVA revealed a difference between two of the four occupational healthcare settings (F = 3.480, *p* = 0.026), with the hospital setting showing a higher mean score (Mean 4.29, SD 0.29, *n* = 13) compared to large companies (Mean 3.92, SD 0.50, *n* = 11) as setting. Similarly, the ANOVA revealed a difference between workers who were screened for Hp (F = 2.81, *p* = 0.040), with healthcare workers showing a higher mean score (Mean 4.27, SD 0.26, *n* = 17) compared to metal workers (Mean 3.79, SD 0.51, *n* = 5). These results suggest that OHCP assessed the delivery of the Hp screening more positively if delivered in hospitals compared to large companies, as well as when delivered to healthcare workers compared to metal workers.

#### Contextual factors

The ANOVA indicated a difference between small & medium-sized enterprises (Mean 4.67, SD 0.01, *n* = 8) and large companies (Mean 4.13, SD 0.39, *n* = 11) within the cccupational healthcare setting (F = 3.53, *p* = 0.024). This suggests that contextual factors within small & medium-sized enterprises were rated slightly more positively compared to large companies. Additionally, the ANOVA revealed a perceived difference among workers who were screened for Hp (F = 4.14, *p* = 0.007). Retail workers showed the highest mean score (Mean 4.83, SD 0.24, *n* = 2), which was higher than that of metal workers (Mean 3.89, SD 0.29, *n* = 5). Metal workers (Mean 3.89, SD 0.29, *n* = 5) and workers in the “Other” category (Mean 4.62, SD 0.08, *n* = 10) also differed, with metal workers scoring lower compared to both retail and other workers. These findings suggest that contextual factors were perceived as slightly more positive if the Hp screening was delivered to retail workers and workers in the “other” category compared to metal workers.

### HCV screening

#### Assessment of the HCV screening

The ANOVA revealed differences in the assessment of HCV screening by OHCP if delivered to healthcare workers (Mean 4.08, SD 0.39, *n* = 22) compared to financial workers (Mean 4.73, SD 0.22, *n* = 5), as well as metal workers (Mean 4.04, SD 0.30, *n* = 5) and financial workers (Mean 4.73, SD 0.22) (F = 5.85, *p* = 0.002). These results suggest that OHCP accessed the HCV screening as slightly more positive if delivered to financial workers compared to healthcare workers or financial workers compared to metal workers.

#### Contextual factors

The ANOVA revealed a difference in contextual factors related to the implementation of HCV screening between hospitals (Mean 4.01, SD 0.72, *n* = 15) and small & medium-sized enterprises (Mean 4.85, SD 0.05, *n* = 8), as well as between large companies (Mean 3.92, SD 0.19, *n* = 10) and small & medium-sized enterprises (Mean 4.85, SD 0.05, *n* = 8) (F = 7.05, *p* < 0.001). This indicates that contextual factors were rated more positively in small & medium-sized enterprises compared to both hospitals and larger companies. Additionally, the ANOVA revealed differences in the influence of contextual factors perceived by OHCP delivering the HCV screening across different workers (F = 4.84, *p* = 0.006). OHCP rated contextual factors as more positive when delivering the HCV screening to “other workers” (Mean 4.85, SD 0.05, *n* = 8), compared to healthcare workers (Mean 4.16, SD 0.68, *n* = 22), financial workers (Mean 3.95, SD 0.13, *n* = 5), and metal workers (Mean 3.88, SD 0.26, *n* = 5). These results suggest variability in how contextual factors were perceived by OHCP delivering the HCV Screening to different worker groups.

### HPV counselling

#### Contextual factors

The ANOVA revealed a difference in contextual factors influencing the implementation of HPV Counselling as perceived by OHCP between larger companies (Mean 3.88, SD 0.26, *n* = 11), hospitals (Mean 4.54, SD 0.52, *n* = 13), and public health institutes (Mean 4.49, SD 0.29, *n* = 9) (F = 9.218, *p* = 0.001), with larger companies showing lower scores compared to both hospitals and public health institutes. These results suggest that the contextual factors within hospitals and public health institutes were perceived as more positive compared to larger companies. Additionally, OHCP perceived differences in delivering HPV Counselling to different workers (F = 10.37, *p* < 0.001), with the highest ratings for healthcare workers (Mean 4.51, SD 0.39, *n* = 13), followed by metal workers (Mean 4.00, SD 0.25, *n* = 5) and financial workers (Mean 3.76, SD 0.21, *n* = 5). These results suggest that OHCP found it more positive or easier to deliver HPV Counselling to healthcare workers compared to metal and financial workers.

## Discussion

The aim of the present study was to examine how OHCPs in Italy, Spain, Romania, and Slovakia assessed the feasibility of three primary cancer prevention programs implemented within the CPW project, namely Helicobacter pylori (Hp) and hepatitis C virus (HCV) screening, and Human Papillomavirus (HPV) counselling and vaccination for workers. Additionally, the study aimed to explore contextual factors influencing the implementation process from the perspective of OHCPs, as well as their perceptions of their roles during the implementation. The findings indicate that integrating primary cancer prevention into occupational health settings is feasible and generally well-received by OHCPs. All three programs were positively evaluated, and organisational conditions, including e.g., resources and leadership support, were perceived as supportive of implementation. OHCP engagement was moderately high, suggesting meaningful participation while highlighting opportunities for further involvement. Assessments varied across settings and worker groups, with more favorable evaluations in hospitals and among healthcare or financial workers compared to other occupational groups. Contextual factors, such as perceived feasibility and ease of implementation, also differed across worker groups, emphasizing the influence of workplace environment and employee characteristics on program delivery.

The consistently high assessments of the three interventions, Hp and HCV screening, as well as HPV counselling and vaccination, suggest provider acceptability and perceived value. These high ratings reflect that OHCPs involved in the implementation and delivery see the prevention programs as both professionally relevant and beneficial to worker health, with perceived benefits outweighing the challenges. Such alignment with clinical values and low perceived disruption reduces the risk of implementation resistance, which often arises when providers anticipate workflow interruptions or negative consequences [27]. Moreover, Damschroder et al. (2022) [19] emphasize that innovations grounded in strong evidence and originating from credible sources are more likely to be successfully implemented [18]. The findings of the present study suggest that OHCPs perceived the prevention programs as evidence-based and originating from reliable sources (see Table 6) — factors which may contribute to greater willingness to support and adopt such interventions in occupational health contexts. Overall, these findings highlight the critical role of evidence-based interventions that align with provider values and workflows in ensuring the successful integration of cancer prevention programs within OHS.

From an epidemiological and public health standpoint, the imperative to scale up preventive programs targeting infection-related cancers is well substantiated. Globally, an estimated 13% of cancers are attributable to chronic infections, Hp and HCV, and HPV collectively accounting for approximately 75% of these cases [6]. In the present study, contextual factors were evaluated mainly positively across all three programs, indicating that OHCPs perceived their organisational environments as largely supportive of implementation. Sufficient time in daily practice, particularly for implementing preventive programs, appeared to enhance the successful implementation of the designed interventions in this study. Additionally, the findings suggest that previous success in introducing new interventions may positively influence the adoption of future programs. According to Damschroder et al.’s framework [27], structural and resource readiness are critical determinants of successful healthcare implementation. Therefore, it is essential to allocate sufficient time within the routine workflow to support the implementation of new interventions and to develop community-based strategies for communication, in addition to individual consultations, to motivate employees and reduce the educational time spent in one-to-one encounters with OHCPs. The findings also indicate support from both occupational healthcare leadership and employers; however, support from local opinion leaders and the organisation itself was somewhat lacking, reinforcing the importance of endorsement across multiple hierarchical levels. Prior research by Aarons et al. [28] demonstrates that transformational leadership enhances implementation by fostering staff motivation and institutional alignment with new initiatives. Additionally, participants perceived the informational materials and program guidance to be well organized and accessible. As emphasized by Greenhalgh et al. [29] the clarity and structure of communication during the diffusion phase significantly enhance adoption, fidelity, and long-term sustainability of health interventions. These findings underscore the necessity of strategic leadership, organisational alignment, and dedicated resources to ensure the successful implementation of infection-related cancer prevention programs within OHS.

In the context of European occupational health systems, routine health surveillance has traditionally focused on exposures to occupational carcinogens [30]. The integration of infection-related cancer prevention into this setting represents a strategic and structural shift toward a more comprehensive approach to worker health [5]. A study conducted by Wiszniewska et al. [30], indicates that over 60% of occupational medicine physicians (OMPs) are willing to implement cancer prevention measures, yet actual engagement remains limited. Older and more experienced OMPs tend to be less proactive, and current practice focuses mainly on obligatory examinations rather than comprehensive prevention [30]. Barriers detected by Wiszniewska et al. [30], include patient consent, communication challenges, and perceptions of workload or responsibility. Strengthening OHCP engagement in activities such as screening and risk assessment is essential for long-term sustainability [19]. These findings somewhat reflect those of the present study: while the integration of infection-related cancer prevention into occupational health services is broadly supported by OHCPs, who largely endorsed the relevance and feasibility of the interventions, their perceived level of engagement in the implementation process was moderate. This suggests that although OHCPs acknowledged involvement in certain stages, there remains substantial room for improvement in fostering deeper and more consistent engagement. On the other hand, many OHCPs in this study did not feel overburdened and generally adapted well to operational changes required for the new programs, supporting the potential for their long-term sustainable implementation. According to Damschroder et al. [19], innovations or new health programs are most effectively implemented when they align with existing workflows. These findings suggest that tailoring prevention programs to local operational contexts and involving OHCPs from the outset is essential for facilitating implementation and enhancing their engagement. OHS remain an underutilized avenue for delivering cancer preventive interventions.

From an implementation science perspective, these findings signal readiness for further scale-up of infection-related cancer prevention programs within occupational health services. The CPW study design, though based on a small-N, descriptive survey, could provide a useful foundation for future interventional studies. While the current study represents an early stage in understanding the potential for involving OHCPs in workplace cancer prevention, it may inform the development of more robust and larger-scale interventions. Moreover, this design could serve as a basis for future research targeting other workplace cancer prevention programs, such as breast and colorectal cancer or melanoma screening, with further refinement and larger sample sizes. Breast cancer screenings could be promoted by rasing awareness about early symptoms, while colorectal cancer screenings, such as regular stool tests and the importance of follow upcolonoscopies after a positive stool test, can be encouraged through awareness campaigns and partnerships with occupational healthcare providers. For melanoma, UV protection education is especially crucial for outdoor workers, and additional awareness could be raised through educational materials emphasizing the importance of regular skin checks by specialists. These screening programs could be effectively integrated into occupational health services during routine check-ups, similar to the preventive programs implemented within the CPW project. The combination of high perceived value and feasibility, along with low perceived harm, creates a favorable climate for institutional adoption. However, attention must be given to minor variability in experience across different settings to ensure consistent engagement and confidence among OHCPs. Future research should systematically investigate scaling strategies for workplace-based cancer prevention programs, with particular focus on contextual feasibility and sustained engagement of OHCPs.

### Strengths and limitations

This study provides unique insights into OHCPs’ perceptions and attitudes toward newly implemented primary prevention programs in Italy, Spain, Romania, and Slovakia within the CPW project. The survey design enabled standardized and comparable data collection across multiple countries. The implementation of the interventions within the occupational setting is novel, offering valuable information for implementation science and policymakers. Limitations include small and uneven sample sizes, which restrict generalisability and prevent detailed country-specific or workplace-specific analysis. As with any survey, results may be influenced by response biases such as social desirability or self-selection, which should be taken into account when interpreting the findings. Both Cronbach’s Alpha and Omega were calculated to assess reliability. However, due to the small sample size and the relatively high standard deviations of the mean scores, these estimates should be interpreted with caution. High variability in the scores may lead to less stable and less accurate reliability coefficients, potentially underestimating the true internal consistency. Therefore, the results should be treated as preliminary, and caution is advised when generalising these findings. No further validation procedures of the mesurments beyond calculation of internal conicteny were performed. It seems that a key limitation of this study is the small overall sample size, as well as the limited number of participants within the various subgroups (e.g., Occupational Healthcare Setting) analysed. This affects the statistical power of the one-way ANOVA tests conducted, potentially limiting the robustness and generalisability of the findings. As a result, differences detected between groups should be interpreted with caution, and future research with larger and more balanced samples is needed to confirm these results. Additionally, the uneven distribution of participants across countries and workplace settings limited the ability to conduct detailed subgroup analyses, which could have provided more nuanced insights. Another limitation could be the small number of resident physicians included in the study. Due to the low participation from this subgroup, a separate analysis of the data was not conducted. Despite these limitations, this study offers valuable preliminary data on OHCPs’ perspectives and highlights areas for further investigation to improve implementation strategies in diverse occupational contexts.

## Conclusion

The baseline OHCP survey suggests that primary prevention programs can be effectively integrated into occupational health services when supported by favorable contextual conditions and active OHCP engagement. While OHCPs generally endorse these programs, increased professional involvement is needed to enhance their long-term impact. Professional involvement refers to actively engaging, taking ownership, and applying expertise to ensure a program is implemented effectively and sustainably. Continuous engagement supports ongoing monitoring and adaptation, helping programs remain responsive to emerging research and evolving health trends. OHCP involvement can be strengthened through targeted training, integration of prevention activities into routine workflows, and allocation of dedicated time and resources to reduce additional workload. Additionally, strong organizational support, interdisciplinary collaboration with specialists, and opportunities for OHCPs to contribute to program design and evaluation further enhance engagement and sustain participation. Tailored, sector-specific strategies may further optimize implementation and sustainability by addressing the unique needs of different occupational settings. Strengthening collaboration between occupational healthcare providers and other stakeholders, such as occupational healthcare managers, external healthcare providers, policy makers, and members of professional associations, will be crucial for maximizing health outcomes. However, scaling such programs across EU workplaces will require both supportive conditions and structured engagement strategies for OHCPs to ensure sustainable integration.

### Implication for practice

To effectively implement primary prevention programs within occupational health services, workplaces should prioritise creating supportive environments for OHCPs. This includes providing tailored training sessions that enhance OHCPs’ knowledge and skills related to infection-related cancer prevention. Clear guidelines and practical toolkits can assist OHCPs in seamlessly integrating new interventions into their routine workflows. Employers and organisational leaders should actively endorse these programs by allocating sufficient time and resources, such as dedicated consultation slots and communication materials for employees. Establishing regular team meetings and feedback channels will help promptly identify and address implementation challenges. Additionally, fostering collaboration between OHCPs, management, and local opinion leaders can strengthen commitment and encourage sustained engagement. By adopting these measures, occupational health services can improve the delivery and sustainability of preventive programs, ultimately benefiting worker health.

## Supplementary Information

Supplementary Material 1. Domains of the second section of the questionnaire.

Supplementary Material 2. Results of the reliability analysis.

## Data Availability

The dataset generated and analysed during the current study will not be made publicly available due to European Data Protection Law but maybe available by the corresponding author on a reasonable request.

## Abbreviations

CFIR: Consolidated Framework for Implementation Reserach
CPW: Cancer Prevention at Work
ERIC: Expert Recommendation for Implementing Change
HCV: Hepatitis C Virus
HPV: Human Papillomavirus
HP: Helicobacter pylori
OHCP: Occupational healthcare professional
OHS: Occupational Health Surveillance
WHO: World Health Organisation
OMP: Occupational Medicine Physicians
