# Supplementary material for: Implementing cancer prevention in occupational healthcare: initial insights from occupational healthcare staff in Central and Southern Europe – findings from the CPW project
Source: BMC Cancer. 2026 Jan 22;26:131. doi: 10.1186/s12885-026-15607-0 (PMC12836969; doi:10.1186/s12885-026-15607-0)
Supplement: Supplementary file 1 — Supplementary Material 1. Domains of the second section of the questionnaire. [file 12885_2026_15607_MOESM1_ESM.pdf]

Supplementary material

Supplementary Material 1: Domains of the Second Section of the Questionnaire

Assessment of the Prevention Programs

1.	The proposed new Screening or Health Education Programs come from a reliable source.
2.	There are many advantages of the new Screening or Health Education Programs with regard to health improvements.
3.	The research evidence for the new Screening or Health Education Programs is sufficiently strong.
4.	The provision of the new Screening or Health Education Programs is sufficiently flexible in practice.
5.	The provision of the new Screening or Health Education Programs is complex in practice.
6.	I see potential harms of the new Screening or Health Education Programs.

Contextual Factors Influencing the Prevention Programs

1.	I have sufficient time in my daily practice to implement the new Screening or Health Education Programs.
2.	My place to work is ready for implementing the new Screening or Health Education Programs (e.g. sufficient facilities for testing).
3.	Information on the new Screening or Health Education Programs for workers is well-organised.
4.	The head of the occupational healthcare team supports the new Screening or Health Education Programs.
5.	The employer(s) support the new Screening or Health Education Programs.
6.	Follow-up after Screening or Health Education Programs of workers (if required) is well-organised.
7.	The new Screening or Health Education Programs have sufficient priority in my place to work.
8.	In previous years, my place to work has generally implemented new Screening or Health Education Programs fast.
9.	Local opinion leaders support the new Screening or Health Education Programs.
10.	The organization provides additional support for implementation of the new Screening or Health Education Programs (e.g. resident physicians).

Role of OHCP During Implementation of the Prevention Programs

1.	My professional attitudes match with the basic principles of the new Screening or Health Education Programs.
2.	From the beginning of the project I feel involved in the implementation of the new screening procedures.
3.	I feel sufficiently competent to implement the new Screening or Health Education Programs.
4.	I can exclude individual workers from the new Screening or Health Education Programs for medical reasons.
5.	I perceive resistance from the workers against the new Screening or Health Education Programs.
6.	Colleagues in my team convinced me to implement the new Screening or Health Education Programs.
7.	I am able to change my operational procedures in daily work to implement the new Screening or Health Education Programs.
8.	I feel overstrained with the changes in relation to the new Screening or Health Education Programs.
9.	The new Screening or Health Education Programs change my area of work/tasks substantially.
