# Supplementary material for: Implementing cancer prevention in occupational healthcare: initial insights from occupational healthcare staff in Central and Southern Europe – findings from the CPW project
Source: BMC Cancer. 2026 Jan 22;26:131. doi: 10.1186/s12885-026-15607-0 (PMC12836969; doi:10.1186/s12885-026-15607-0)
Supplement: Supplementary file 2 — Supplementary Material 2. Results of the reliability analysis. [file 12885_2026_15607_MOESM2_ESM.pdf]

Supplementary material

Supplementary Material 2: Results of the Reliability Analysis

	N	Number of items	Answering categories	Scale construction	Cronbach's alpha	Omega
**Assessment of the Prevention Programs**						
HCV Screening	37	6	5	Average	0.41	0.88
HP Screening	36	6	5	Average	0.53	0.83
HPV Counselling	22	6	5	Average	0.27	0.84
**Contextual Factors Influencing the Prevention Programs**						
HCV Screening	18	10	5	Average	0.92	0.95
HP Screening	16	10	5	Average	0.75	0.88
HPV Counselling	13	10	5	Average	0.84	0.90
**OHCP Role During Implementation of the Prevention Programs**						
HCV Screening	24	9	5	Average	0.57	0.76
HP Screening	35	9	5	Average	0.61	0.76
HPV Counselling	15	9	5	Average	0.57	0.84
